# Influence of Soybean Cultivar Resistance on the Foraging Behavior of *Encarsia* sp. Against *Bemisia tabaci* MEAM1

**DOI:** 10.1002/arch.70092

**Published:** 2025-09-15

**Authors:** Maria Carolina Farias e Silva, Raylson Lopes da Silva, Matheus Monteiro de Santana, Daniel Marques Pacheco, Jean Pierre Cordeiro Ramos, Rafael de Souza Miranda, Jose Bruno Malaquias, Jenilton Gomes da Cunha, Bruno Ettore Pavan, Luciana Barboza Silva

**Affiliations:** ^1^ Graduate Program in Agricultural Sciences Federal University of Piauí Bom Jesus Brazil; ^2^ Department of Agronomy Federal University of Piauí Bom Jesus Brazil; ^3^ Entomology Laboratory, Center for Agrarian Sciences Universidade Federal da Paraíba Areia Brazil; ^4^ Department of Plant Science, Food Technology and Partner Economy Júlio de Mesquita Filho Paulista State University Ilha Solteira Brazil

**Keywords:** *Glycine max*, herbivore‐induced volatiles, host resistance, olfactometer, parasitoid foraging, whitefly

## Abstract

*Bemisia tabaci* MEAM1 is a significant pest in soybean crops, posing a challenge for control and requiring novel strategies within Integrated Pest Management (IPM). This study evaluated the host‐searching behavior of the parasitoid *Encarsia* sp. in response to volatiles emitted by soybean cultivars infested with *B. tabaci*. Using a Y‐shaped olfactometer, we tested the cultivars BRASMAX BÔNUS IPRO® (susceptible) and M 8808 IPRO® (resistant by antixenosis), assessing the parasitoid's response to the emitted volatiles. Additionally, we conducted behavioral bioassays to evaluate *Encarsia* sp. exploration on leaf disks of the infested cultivars. The results showed that the parasitoid spent more time in the central region of the olfactometer, with no clear preference between cultivars. However, in the foraging bioassays, *Encarsia* sp. explored the susceptible cultivar more actively, demonstrating a higher frequency of interactions such as antennation and attack on nymphs. The resistant cultivar exhibited lower behavioral diversity, suggesting reduced chemical or physical stimulation for the parasitoid. These findings indicate that the intrinsic characteristics of soybean cultivars can enhance the effectiveness of biological control and constitute a key factor in IPM strategies aimed at optimizing parasitoid performance in whitefly control.

## Summary

- Foraging behavior of *Encarsia* sp. assessed across soybean cultivars with differing resistance to *Bemisia tabaci* MEAM1.
- Soybean plant volatiles did not attract *Encarsia* sp. in Y‐tube olfactometer assays.
- In leaf‐disc arenas, *Encarsia* sp. showed higher activity on the susceptible cultivar.
- Findings indicate cultivar resistance can modulate parasitoid host‐searching cues.
- Results inform strategies to enhance *Encarsia*‐mediated whitefly biocontrol in IPM.

## Graphical abstract

The volatiles emitted by tested soybean cultivars did not significantly affect the attraction of *Encarsia* sp. parasitoid in Y‐shaped olfactometer. The cultivar Bônus may exert a more attractive effect and diversified foraging behavior compared to whitefly‐resistant M8808 cultivar.

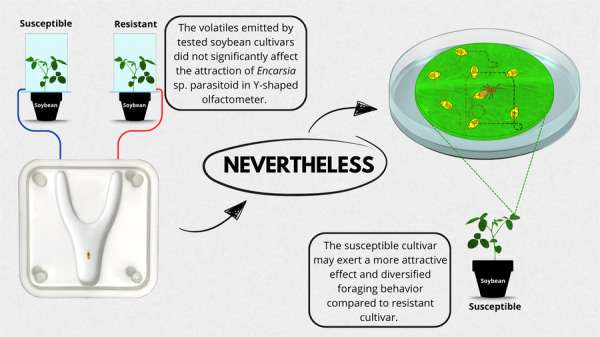

## 1 Introduction

*Bemisia tabaci* (Hemiptera: Aleyrodidae) biotype B, or Middle East‐Asia Minor 1 (MEAM1), known as whitefly, has become a key pest in soybean crops, causing significant economic losses in several countries, including Brazil (Lima et al. 2002; Pozebon et al. 2020). Studies indicate that just one whitefly per trifoliate can reduce soybean productivity by up to 31.24 kg per hectare (Padilha et al. 2021). Over the past decade, *B. tabaci* control has become increasingly difficult, with recurrent outbreaks in soybean fields in Brazil (Dângelo et al. 2018; Arnemann et al. 2019; Pozebon et al. 2020; Almeida et al. 2021). This situation is also recurrent in Bom Jesus city, Piauí state, at Brazil country, an area of agricultural fron‐tier expansion that comprises a region known as MATOPIBA (an acronym formed from the initials of the four states: Maranhão, Tocantins, Piauí, and Bahia) (Rufo et al. 2020). This region of intense soybean production suffers from the damage caused by *B. tabaci* MEAM1, requiring the urgent development of new strategies within Integrated Pest Management (IPM) programs to mitigate these impacts (Bale et al. 2008; Vandervoet et al. 2018).

IPM using the parasitoid *Encarsia* sp. is a promising tool for controlling *B. tabaci*. The genus *Encarsia*, which belongs to the Aphelinidae family, includes several species effective for the biological control of *B. tabaci* (Stansly and Naranjo 2010). In sweet potato, *Encarsia tabacivora* was found to parasite *B. tabaci* nymphs, helping to mitigate viral populations (Gamarra et al. 2023). *Encarsia sophia* has also demonstrated efficacy in reducing *B. tabaci* populations through parasitism and direct host feeding, especially under high pest density conditions (Katono et al. 2022). *Encarsia formosa* is one of the most studied species, widely known for its nonconsumptive effects, which reduce the fecundity and longevity of *B. tabaci*, making it a standout biological control agent in protected cultivation systems and open fields (Fan et al. 2021).

The foraging behavior of natural enemies is strongly influenced by volatile organic compounds (VOCs) emitted by plants attacked by herbivores, known as Herbivore‐induced plant volatiles (HIPVs). These volatiles are released in response to herbivore attacks and vary according to the plant species, genotype, developmental stage, and various environmental and biotic factors (Clavijo McCormick et al. 2012). HIPVs act as chemical signals that help natural enemies locate infested plants and potential hosts (Turlings and Erb 2018). The most common compounds include terpenes, esters, and alcohols, which are known to be effective in attracting parasitoids (Hare 2011). The use of HIPVs as a biological control strategy has been studied with a focus on retaining natural enemies within crops, potentially improving agricultural sustainability and reducing pesticide use (Turlings and Erb 2018; Kaplan 2012; Ali et al. 2023).

HIPVs have been shown to effectively attract *Encarsia* and other parasitoid wasps in several plant species, such as tomato, pepper, melon, and cotton (Zhu and Park 2005; Gurr et al. 2011; Chen et al. 2020). These compounds are released by both susceptible and resistant cultivars and can be manipulated to optimize biological control in crops such as soyean (Vet and Dicke 1992). Recent studies have advanced the understanding of the specificity of these interactions (Ayelo et al. 2021; Marmolejo et al. 2021; Waterman et al. 2024); however, the interactions mediated by HIPVs in soybean cultivars under *B. tabaci* MEAM1 infestation remain poorly explored, especially under semiarid environmental conditions like those found in northeastern Brazil.

In this context, studying the behavior of new natural enemies is essential to assess their potential as biological control agents (Moro et al. 2021), especially considering that each species may exhibit specific preferences for hosts or host plants (Arikan et al. 2024). The use of natural enemies native to the region should be prioritized, as they are already adapted to local environmental conditions, enhancing their efficacy in pest suppression and reducing risks to native ecosystems. A better understanding of how these organisms interact with their hosts and plants enables the development of more sustainable and effective IPM strategies, reducing reliance on chemical insecticides and promoting ecological balance in agricultural systems.

Our investigative study aimed to evaluate the search and foraging behavior of Encarsia sp. in response to HIPVs emitted by susceptible and resistant soybean cultivars under infestation by *B. tabaci* MEAM1. Understanding these tri‐trophic interactions between plant, herbivore, and parasitoid is necessary to improve biological control in soybean cultivation systems, providing subsidies to mitigate the damage caused by *B. tabaci*, as well as the development of new tools for IPM.

## 2 Materials and Methods

### 2.1 Site of the Experiment

The experiment was conducted in the Plant Propagation Laboratory of the Federal University of Piauí – Professora Cinobelina Elvas Campus (Latitude 9°05′02.9″ S; Longitude 44°19′34.2″ W; and Altitude 270), in a controlled environment (35°C ± 2; 60%–70% RH), in the city of Bom Jesus, Piauí, Brazil. It took place from January to June, during the rainy season in the region. The trial was exposed to natural photoperiods typical of this period in the Southern Hemisphere, with approximately 13 h of daylight and 11 h of darkness per day.

### 2.2 Cultivars

The cultivars of soybean were chosen based on previous studies of the history of resistance and susceptibility to *B. tabaci* MEAM1 (Rodrigues et al. 2021a, 2021b; Silva et al. 2023), described in Table 1.

**Table 1:** Soybean cultivars [*Glycine max* (L.) Merill, Fabacea] used and their respective responsible company and history of resistance to *Bemisia tabaci* MEAM1.

Cultivars	Company	Resistance kind register
BRASMAX BÔNUS IPRO®	Brasmax	Susceptible (Rodrigues et al. 2021b; Silva et al. 2023)
M 8808 IPRO®	Monsoy	Antixenosis and Antibiosis (Rodrigues et al. 2021b; Silva et al. 2023)

Soybean cultivars were treated with insecticide and fungicide (Dermacor® BR + Spectro®) and sown in plastic pots (5 L, 18 cm high, 22 cm upper diameter, and 16 cm lower diameter), filled with 5 kg of autoclaved substrate consisting of soil (argisolic dystrophic yellow latosol) and organic matter (autoclaved, cured cattle manure) in a 3:1 ratio. The sub‐strate was fertilized according to recommendations based on soil analysis interpretation (Sousa and Lobato 1996). The plants were maintained in a greenhouse at 35°C ± 2, 60%–70% relative humidity, and kept free from *B. tabaci* infestation.

### 2.3 Rearing of the *B. tabaci* Population

Whitefly nymphs (*B. tabaci*) were initially collected from an insecticide‐free tomato crop and taken to the laboratory to establish a colony. For continuous rearing, cabbage plants (*Brassica oleracea* L. var. sabellica) were used as the host. These plants were grown in five‐liter pots filled with a substrate composed of soil, washed sand, and cattle manure in a 1:1:1 ratio. The pots were kept in a greenhouse whose roof was covered with shade cloth and transparent canvas, while the sides were enclosed with a 50‐mesh antivenom screen. The plants were monitored daily to remove aphids and caterpillars and fertilized according to soil analysis recommendations for the crop. To confirm the identity of the *B. tabaci* biotype used in the study, adult specimens were sent to Embrapa Arroz e Feijão for molecular identification.

### 2.4 Rearing of the *Encarsia* sp. Population

The parasitoid rearing was adapted according to the methodology proposed by Generoso (2006). The initial population of *Encarsia* sp. was obtained from the rearing of *B. tabaci* established at UFPI, Campus Professora Cinobelina Elvas, in the municipality of Bom Jesus‐PI, through the collection of *B. tabaci* nymphs on kale plants. For multiplication of the parasitoids, kale plants containing *B. tabaci* nymphs were subjected to parasitism by *Encarsia* sp.

Specimens were sent to the Biological Institute for taxonomic analysis and species identification. The identification was performed based on morphological characteristics and molecular analysis, following standard protocols for the genus *Encarsia*. The results did not match any described species, suggesting the existence of a potentially new species. The specimens are currently undergoing detailed characterization for formal description and taxonomic confirmation.

### 2.5 Behavior of *Encarsia* sp. in Response to Soybean Volatiles Under *Bemisia tabaci* Herbivory Using a Y‐Shaped Olfactometer

Soybean plants of each cultivar (M 8808 IPRO and BRASMAX BÔNUS IPRO) in the V4 vegetative stage (four branches of compound leaves after the cotyledons) were used for the experiment. The plants were placed in voile cages and infested individually with 100 non‐sexed adult insects of *B. tabaci*. After the 8‐day infestation period, the adult insects were removed from the plants, leaving only the nymphs and eggs. The plants were taken to the laboratory, and the behavior of the parasitoid *Encarsia* sp. was evaluated in an olfactometer bioassay.

**Figure 1:**
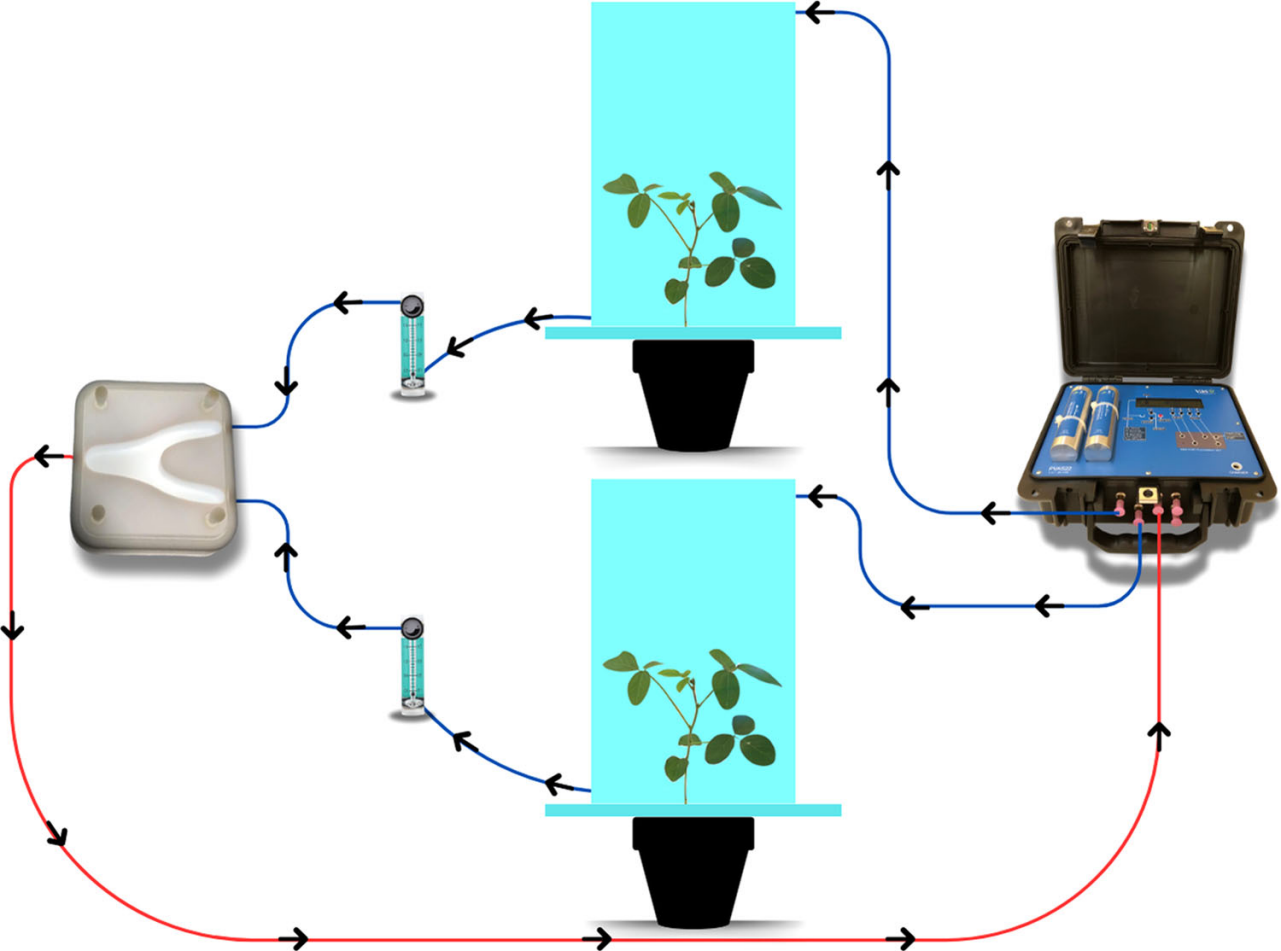
Schematic of the olfactometer system used in the bioassay. The blue lines indicate the hoses that transport the purified air to the chambers containing the treatment plants, allowing the collection of volatiles. The airflow then goes to the Y‐shaped olfactometer, where the behavior of the parasitoid is evaluated. The red line represents the air flow removed from the Y‐shaped olfactometer, ensuring continuous circulation within the system. The pump (on the right) purifies and controls the air flow.

To verify the interference of volatiles released by soybean plants infested with *B. tabaci* on the behavior of the parasitoid *Encarsia* sp., a Y‐shaped olfactometer was used. The olfactometer consists of an acrylic plate with a hole in the center, in the shape of a Y, with a central body measuring 5.0 × 2 cm and two arms measuring 5.0 × 2 cm each. The olfactometer was closed at the top by a glass plate with four fine screws, ensuring the sealing of the internal area. The air was driven into the olfactometer by a PVAS22 pump (Volatile Assay Systems, Rensselaer, NY, USA). Before entering the system, the air was filtered by hydrocarbon filters already attached to the pump. Subsequently, the air was conducted through PTFE tubes regulated at (2.0 L/min) to each acrylic chamber (with dimensions of 0.50 × 0.30 cm, with a centralized hole of 19 cm in diameter) where the plants were placed individually. Before placing the aerial part of the plant in the acrylic chamber, the soil of the pots was sealed with aluminum foil, ensuring air circulation inside. After passing through the plants, the air was conducted through polyethylene hoses, regulated by a flowmeter at (0.5 L/min), to the arms of the olfactometer (Figure 1).

Ninety replicates of each treatment combination were performed: (i) M 8808 IPRO® vs EMPTY CHAMBER; (ii) BRASMAX BÔNUS IPRO® vs EMPTY CHAMBER; and (iii) M 8808 IPRO® vs BRASMAX BÔNUS IPRO®, totaling 270 replicates. Each replicate consisted of one female *Encarsia* sp. Each female was individually introduced into the Y‐shaped olfactometer, positioned in the release area. To minimize possible influences on the insect response, the position of the treatments was alternated every three bioassays. Furthermore, the plants were replaced every six bioassays, avoiding pseudoreplication problems. The design was completely randomized with three treatments and ninety replicates.

Insect behavior was recorded for 10 min using a filming system consisting of a 4 K UHD webcam (EMEET SmartCam S600) installed directly above the center of the arena (olfactometer). Videos were recorded using OBS Studio (Open Broadcaster Software) and processed using EthoVision XT motion tracking software. The variables measured were: (1) Cumulative duration (%) (proportion of time the insect spent in each zone, allowing a percentage analysis of exploratory behavior concerning the olfactometer zones); (2) Average speed (cm/s) (The average speed of displacement was calculated for each insect. This parameter provides information on the intensity of movement in response to olfactory stimuli and allows inferring the degree of activity in the olfactometer); (3) Total Distance Traveled (cm) (The total distance traveled was measured throughout the olfactometer arena. This variable reflects the insect's locomotion activity and may indicate its level of exploration in the arena); (4) Not Moving (proportion of time the insect remained still in the arena). Heat maps were also generated, which allowed us to identify, through color patterns, the insect's permanence in the Y olfactometer arena.

### 2.6 Bioassay of Foraging Behavior of *Encarsia* sp. in Soybean Cultivars Under Herbivory of *Bemisia tabaci*

Soybean cultivars from the previous bioassay were used to evaluate the foraging behavior of *Encarsia* sp. The plants were infested with 50 individuals of *B. tabaci*, using voile cages (13 × 13 cm) on two leaves of each plant. After 24 h, the whitefly adults were removed. Eleven days after infestation, when the nymphs reached the third and fourth instar stages, leaves containing nymphs were cut into 1 cm diameter disks. The disks were fixed on plates (arenas) measuring 3.5 cm in diameter and 1 cm in height, filled with 2% agar to prevent the leaf disks from wilting.

**Figure 2:**
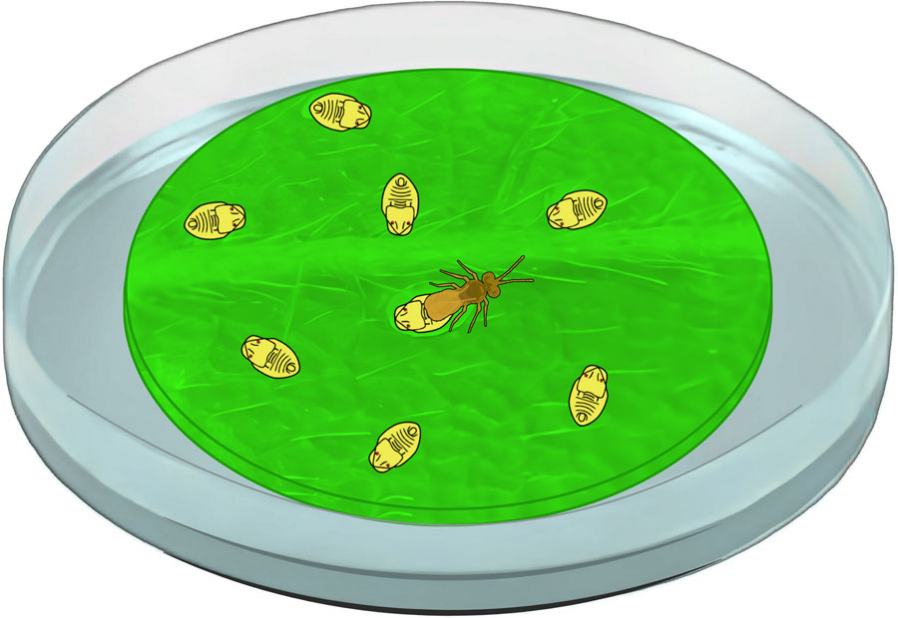
Illustration of a Petri dish containing a soybean leaf disc infested with *Bemisia tabaci* MEAM1 nymphs. In the center, a female *Encarsia* sp. can be seen, used to evaluate its foraging behavior and parasitism on the nymphs.

To record foraging behavior, a female *Encarsia* sp. parasitoid was placed in each arena (Figure 2). The behavior was captured using a 4 K UHD webcam (EMEET SmartCam S600). Videos were recorded using OBS Studio software (Open Broadcaster Software). Recording started as soon as the parasitoid positioned itself on the leaf disc and began to move, but it stopped when the insect left the leaf disc. The bioassay had 20 replicates for each soybean cultivar, totaling 40 experimental units. The design was completely randomized, with two treatments and twenty replicates.

Based on observations, a list of foraging behaviors (Table 2) of *Encarsia* sp. on *B. tabaci* was prepared. These behaviors were used to create ethograms. We followed the procedure described by Ramirez‐Romero et al. (2012) and Velasco–Hernández et al. (2013) to analyze behavioral sequences. The results of the behavioral sequences were represented graphically using ethograms.

**Table 2:** Catalog of behaviors of *Encarsia* sp. was analyzed in this study.

Event	Description
Walk	The parasitoid moves on the surface of the leaf disc.
Rest	The parasitoid remains immobile.
Groom	Grooming any body part, including stroking the antennae, ovipositors, or wings.
Antennation	The parasitoid touches the nymphs with its antennae.
Tarsi	The parasitoid touches the nymph with the tarsus.
Drag	The parasitoid rubs its ovipositor on the leaf surface.
Attack	The parasitoid inserts the ovipositor inside the nymph.

### 2.7 Statistical Analysis

The data from the olfactometer bioassay were subjected to generalized linear model (GLM) test to verify the fit to different distributions, according to the nature of the response variable. When no model fit, nonparametric tests were adopted. Comparisons between zones were performed using the Wilcoxon test, while the Kruskal‐Wallis test assessed differences between treatments and means by the Dunn test with Bonferroni correction (*p* < 0.05). Statistical analyses were performed using R software (R Core Team 2024). Heat maps were generated using the Ethovision program, which overlaps 45 replicates of each treatment. A color scale was created: zones where the insect spent more time in red and blue tones and where the insect spent less time.

In the foraging behavior bioassay, GLM was performed to find significant transitions within each cultivar and between cultivars. The Krukal‐Wallis test was used when no model was adjusted. Means were calculated using the Dunn test with Bonferroni correction (*p* < 0.05). All analyses were performed using R software (R Core Team 2024).

## 3 Results

### 3.1 Behavior of *Encarsia* sp. in Response to Soybean Volatiles Under *Bemisia Tabaci* Herbivory Using a Y‐Shaped Olfactometer

**Figure 3:**
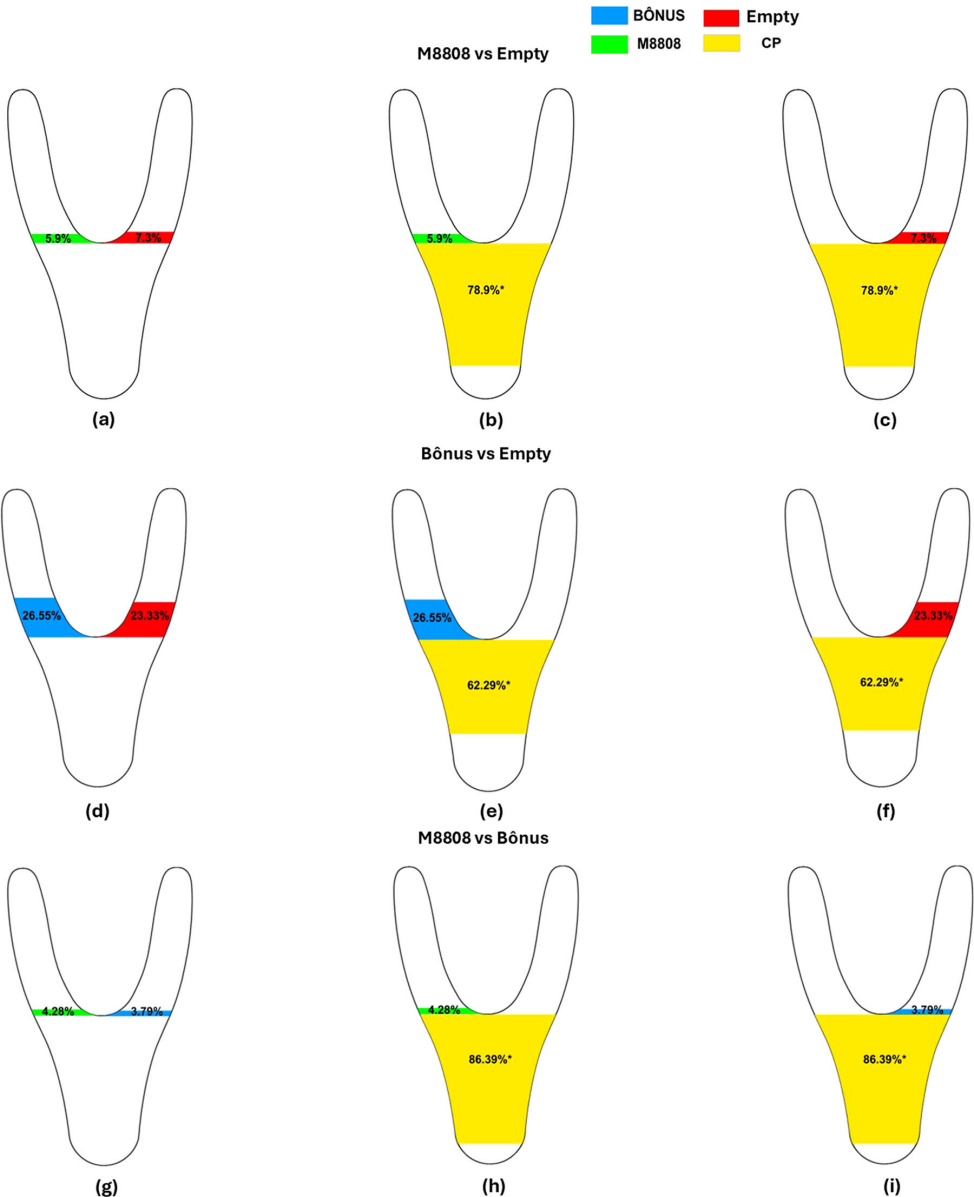
Distribution of cumulative duration (%) of Encarsia sp. permanence in different zones of the Y‐shaped olfactometer connected to chambers containing soybean cultivars infested with *Bemisia tabaci* MEAM1. Three combinations were evaluated: M8808 versus empty (a–c), Bonus versus empty (d–f), and M8808 versus Bonus (g–i). The colors represent the different zones of the arena: blue (arm with the Bonus cultivar), green (arm with the M8808 cultivar), red (empty arm), and yellow (center of the arena). Values marked with an asterisk (*) indicate significant differences by the Wilcoxon test (*p* < 0.05).

The analysis of the cumulative duration (%) of *Encarsia* sp. permanence in different zones of the Y olfactometer revealed distinct spatial responses depending on the treatments evaluated (Figure 3). In the bioassays comparing the M8808 cultivar with the empty chamber (Figure 3a–c), the parasitoids showed a significant preference for the central zone (CP) (represented in yellow; 78.9%) compared to the empty zone (red; 7.3%) and the M8808 zone (green; 5.9%), as indicated by asterisks (*, *p* < 0.05). Similarly, in the comparison between the Bônus cultivar and the empty chamber (Figure 3d–f), *Encarsia* sp. spent a substantially greater proportion of time in the central zone, where volatiles from the Bônus cultivar (blue) were present.

In the direct comparison between the M8808 and Bônus cultivars (Figure 3g–i), the data showed that the parasitoids spent more time in the central zone than in the arms associated with either cultivar (Wilcoxon test, *p* < 0.05). This pattern suggests no distinct attractiveness of the volatiles emitted by either cultivar.

**Figure 4:**
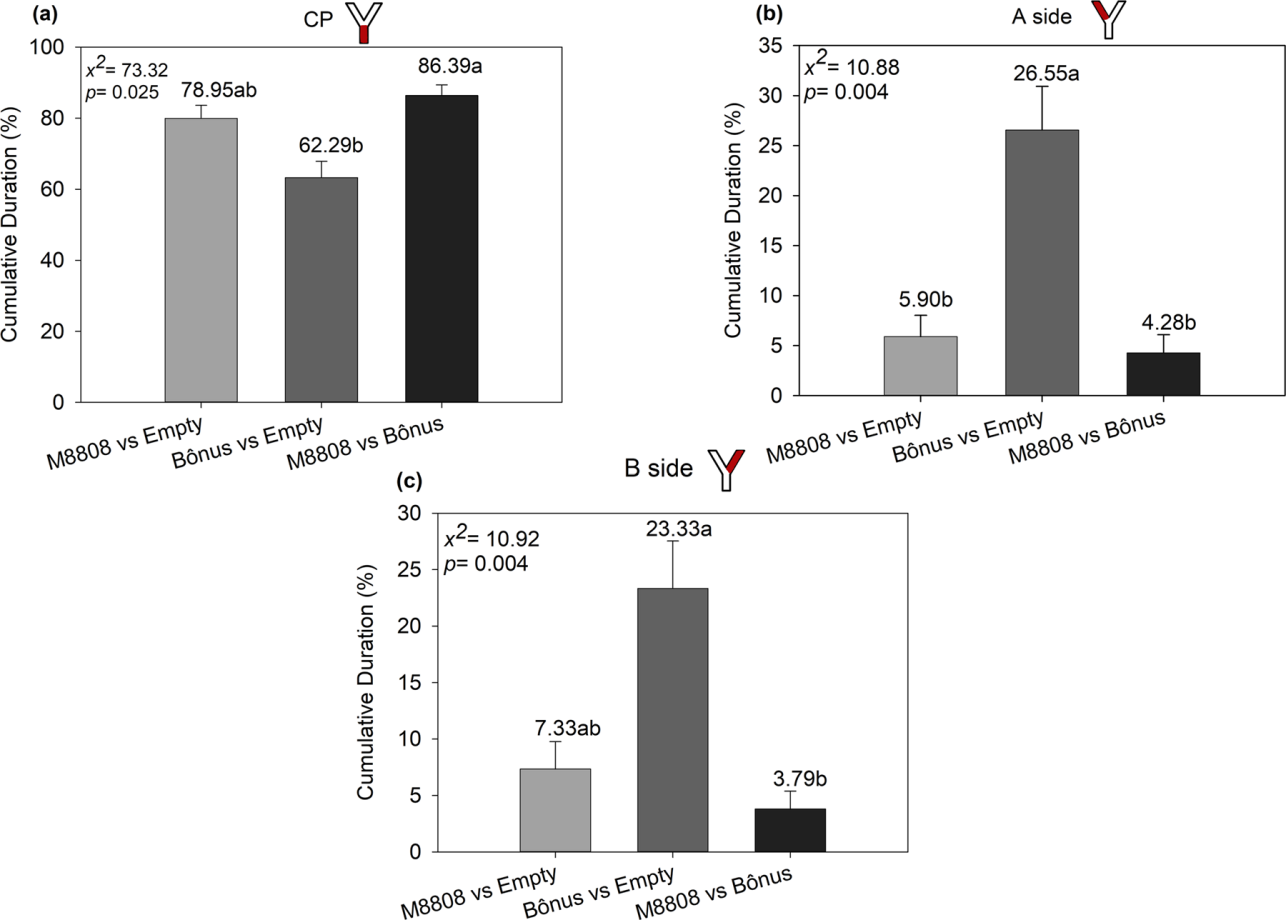
Cumulative duration (%) of *Encarsia* sp. residence time in response to volatiles emitted by different soybean cultivars infested with *Bemisia tabaci* MEAM1, evaluated in a Y‐shaped olfactometer. The analyses compare the treatments M8808 versus Empty, Bonus versus Empty, and M8808 versus Bonus in the following regions of the olfactometer: (a) residence time in the central region of the Y (CP), (b) residence time in zone A of the Y and (c) residence time in zone B of the Y. Equal letters indicate that the treatments do not differ statistically from each other by the Dunn test with Bonferroni correction (*p* < 0.05).

The cumulative duration (%) of *Encarsia sp.* residence time differed significantly when compared to the different zones among treatments (Figure 4). In the center of the Y (CP) (Figure 4a), *Encarsia sp.* remained longer in M8808 vs. Bonus (86.39%) than in Bonus vs. Empty (78.95%) and M8808 vs. Empty (62.29%). In zone A (Figure 4b), permanence was higher in Bônus vs. Empty (26.55%) compared to M8808 vs. Empty (5.90%) and M8808 vs. Bônus (4.28%). In zone B (Figure 4c), Bônus vs. Empty (23.33%) differed from M8808 vs. Bônus (3.79%), while M8808 vs. Empty (7.33%) had an intermediate value. These results indicate that the volatiles of the evaluated cultivars influence the behavior of *Encarsia sp.*, favoring its permanence in the presence of the Bônus cultivar infested by B. *tabaci*.

**Figure 5:**
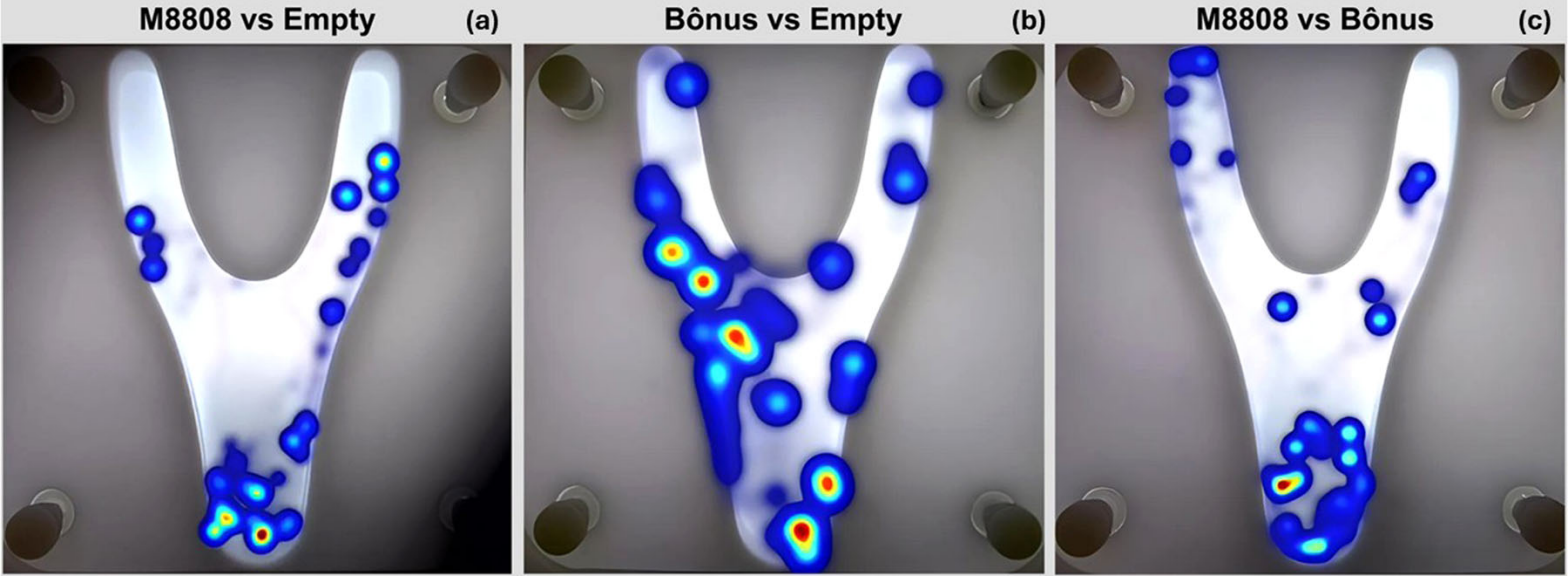
Heat map of Encarsia sp. permanence in the Y‐shaped olfactometer in three comparisons: (a) soybean cultivar M8808 infested with *Bemisia tabaci* versus Empty, (b) Bônus cultivar infested with *B. tabaci* versus Empty, and (c) M8808 versus Bônus. Each trial lasted 10 min, totaling 15 h per treatment. The color of a pixel represents the total time that *Encarsia* sp. remained in that zone, with warmer colors denoting more time spent. Heat maps were generated using EthoVision software to track individuals’ movement.

The heat maps obtained by tracking the parasitoid *Encarsia* sp. in the Y‐shaped olfactometer show differences in individual permanence between treatments (Figure 5). In the comparison between the soybean cultivar M8808 infested with *B. tabaci* and the empty arena (Figure 5a), a dispersed distribution of the parasitoid was observed, with greater permanence in the lower region and some concentration in the upper right end of the olfactometer, suggesting a preference for the empty zone. In the comparison between the in‐fested cultivar Bônus and the empty zone (Figure 5b), a distinct pattern emerged, with a higher concentration of parasitoids near the bifurcation leading to the infested cultivar, indicating zones of greater permanence and higher attractiveness to the Bônus cultivar. In the direct comparison between the infested cultivars M8808 and Bônus (Figure 5c), the parasitoids remained predominantly in the center of the olfactometer, indicating no clear attraction to either cultivar. These results suggest that parasitoid attractiveness varies depending on the soybean cultivar.

**Figure 6:**
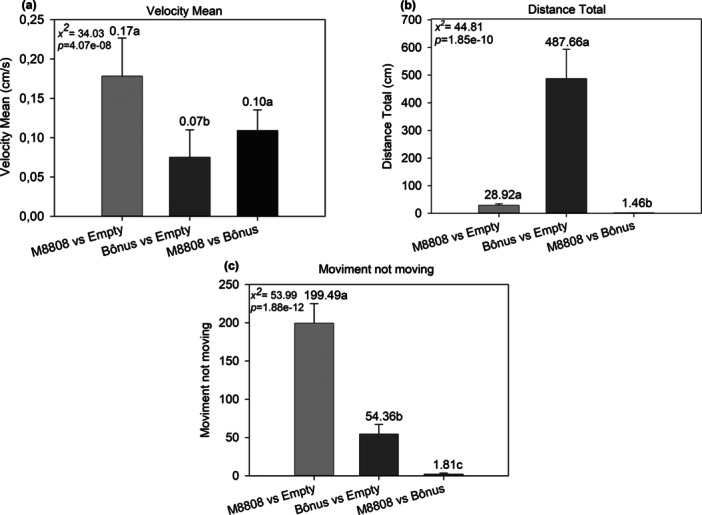
Displacement parameters of *Encarsia* sp. in Y‐olfactometer. (a) Average displacement speed (cm/s), (b) total distance traveled (cm), and (c) time without movement (s), connected to chambers containing different treatments: M8808 versus Empty, Bonus versus Empty, and M8808 versus Bonus. Equal letters indicate that the treatments do not differ statistically by the Dunn test with Bonferroni correction (*p* < 0.05).

The average movement speed of *Encarsia* sp. (Figure 6a) was significantly higher in the treatments M8808 versus Empty (0.17 cm/s) and M8808 versus Bonus (0.10 cm/s) compared to Bonus versus Empty (0.07 cm/s). The total distance traveled (Figure 6b) was significantly greater in the Bonus versus Empty treatment (487.68 cm) than in M8808 versus Empty (28.92 cm) and M8808 versus Bonus (54.80 cm), which did not differ from each other. Regarding the time without movement (Figure 6c), M8808 versus Empty showed the longest duration (199.43 s), followed by Bonus versus Empty (54.36 s) and M8808 versus Bonus (1.81 s). These results indicate that the volatiles emitted by the cultivars influence specific behavioral aspects of *Encarsia* sp. in the olfactometer—particularly the distance traveled and the time spent inactive—demonstrating differentiated behavioral responses depending on the treatment.

### 3.2 Bioassay of Foraging Behavior of *Encarsia* sp. in Soybean Cultivars Under Herbivory of *Bemisia tabaci*

The analysis of *Encarsia* sp.'s behavioral transitions when foraging on leaf disks of the soybean cultivars Bônus and M8808 revealed distinct behavioral patterns (Figure 5). The most frequent behaviors for both cultivars were walking, grooming, and resting; however, the frequency and transitions between these states varied across treatments.

**Figure 7:**
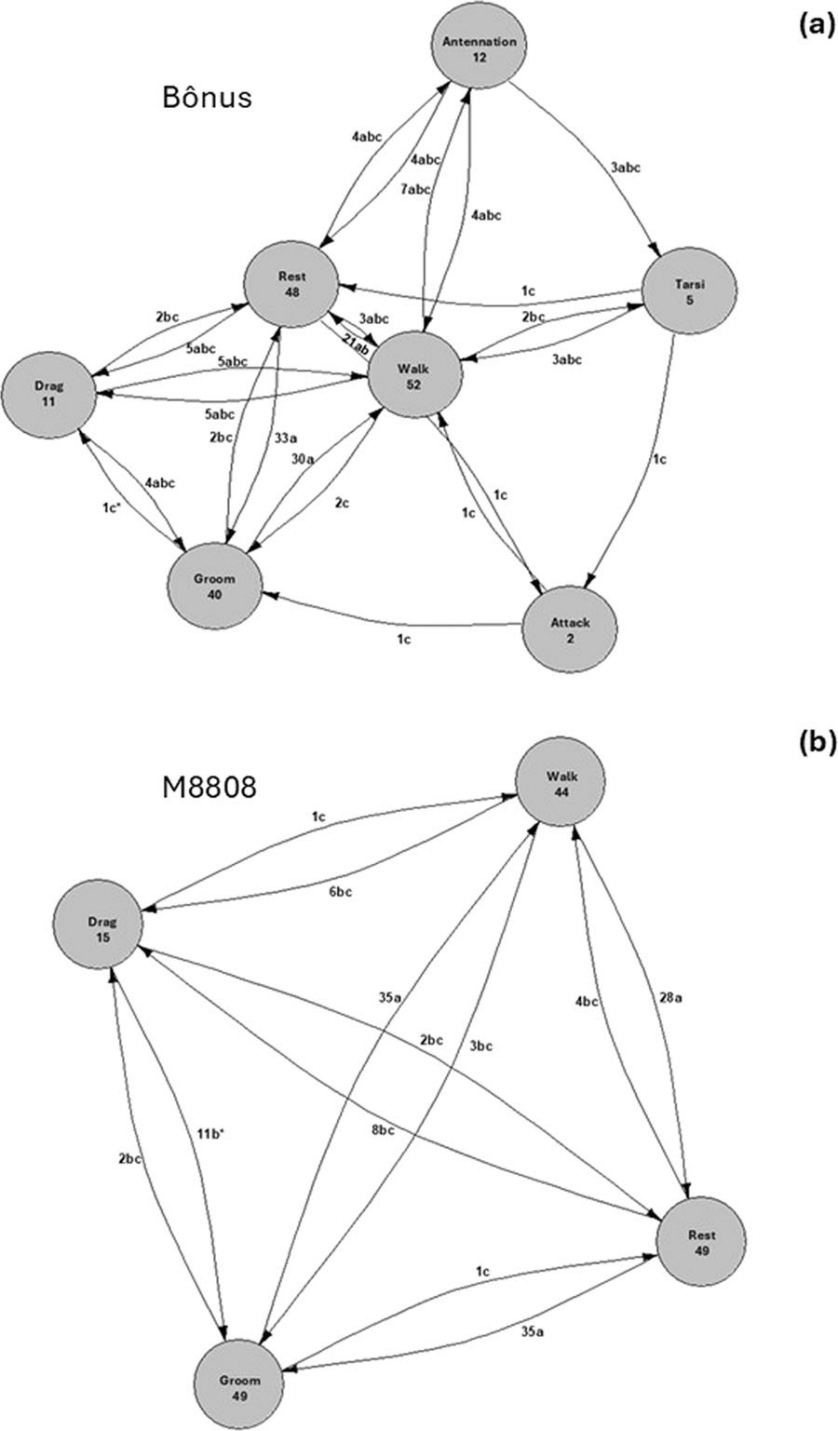
Ethograms representing behavioral transitions of Encarsia sp. when foraging on leaf disks of soybean cultivars Bônus (a) and M8808 (b) infested with nymphs of *Bemisia Tabaci* MEAM1. The nodes represent different behaviors, while the arrows indicate the transition between them. The numbers next to the arrows correspond to the frequency of the observed transitions. Equal letters indicate that the treatments do not differ statistically by the Dunn test with Bonferroni correction (*p* < 0.05). Asterisks (*) indicate significant differences between treatments.

In the Bônus cultivar (Figure 7a), walking behavior showed high connectivity, with frequent transitions to resting, grooming, and antennation. Additionally, greater diversification was observed in behavioral transitions, including interactions with the tarsi, dragging, and attacking behaviors, suggesting more active exploration of the substrate.

In the M8808 cultivar (Figure 7b), walking behavior was still central but showed fewer transitions. The interactions were mainly directed toward resting, grooming, and dragging, indicating a more linear and less diverse exploration pattern than in the Bônus cultivar. Notably, transitions to antennation and attack in this cultivar were less prominent, suggesting possible differences in substrate attractiveness and parasitoid behavioral response.

## 4 Discussion

Natural enemies locate their hosts through three foraging sequences involving habitat location and host selection, mainly using combinations of long‐range, short‐range, and contact chemical cues (Fatouros et al. 2008). The bioassay performed in the olfactometer indicated that *Encarsia* sp. did not show a clear and differentiated attraction to the volatiles of the M8808 and Bônus cultivars infested with *B. tabaci* MEAM1. The time spent by the parasitoid in the center of the Y arena was significantly longer than in the arms when comparing the zones within the treatments. However, the results of the time spent by *Encarsia* sp. between treatments and the heatmaps show a tendency of the insect towards the Bônus cultivar.

The olfactometer's absence of a directed response suggests that *Encarsia* sp. may not use cues associated with *B. tabaci* MEAM1 nymphs to guide its search for the host. These results are similar to studies by Chen et al. (2020), who demonstrated that volatiles induced by *B. tabaci* infestation in tomato plants did not attract *Encarsia formosa* to nymphs but rather to whitefly adults. The findings indicate that parasitoids may rely more on chemical cues emitted by adults to locate their hosts, while nymphs possibly do not generate sufficiently distinct chemical stimuli to attract parasitoids over long distances. The presence of other plant stressors, such as plant pathogen viruses, may also interfere with and amplify the production of volatiles attractive to *Encarsia* sp. (Pan et al. 2013; Liu et al. 2014; Liu et al. 2018).

*Encarsia* sp. showed a higher average displacement speed in the M8808 versus Empty and M8808 versus Bônus treatments, while the greatest distance traveled was observed in the Bônus versus Empty treatment. These results indicate that the Bônus cultivar may provide an environment more actively explored by the parasitoid, while M8808 may lead to a more cautious or less exploratory behavior. However, the results of the foraging experiment on the leaf disc demonstrated that *Encarsia* sp. adjusts its foraging behavior according to the cultivar, especially in the diversity of behavioral transitions and substrate exploration. In the foraging of the Bônus cultivar, there was a greater diversity of behaviors and transitions. The parasitoid had a higher frequency of antennation and attack, suggesting a higher level of exploration and interaction with the substrate. In the M8808 cultivar, the behavioral transitions were more restricted, with a predominance of walking, grooming, and resting and fewer exploratory interactions. This may indicate less chemical or physical stimulation for parasitoids, resulting in less active exploration.

Differences in foraging behavior among cultivars may be associated with morphology and chemical composition. Previous studies suggest that resistant cultivars, such as M8808, may present compounds that reduce host acceptance by whitefly, either by altering the nutritional quality of the nymphs or due to low nymph infestation levels on the leaves (Rodrigues et al. 2021b; Silva et al. 2023). Studies by Katono et al. (2022) and Arikan et al. (2024) demonstrate that parasitoids of the genus *Encarsia* are more active in parasitism at higher densities of *B. tabaci* nymphs. In addition, the plant's physical characteristics, such as trichome density and leaf texture, may influence the efficiency of parasitoids in locating their hosts (Ayelo et al. 2021).

Leaf disks of the Bônus cultivar were observed to contain a greater amount of honeydew, a sugary substance produced during the feeding of *B. tabaci* nymphs. The honeydew likely contributed to the more active behavior of *Encarsia* sp., as honeydew volatile compounds are potentially attractive to the parasitoid (Ayelo et al. 2022).

Our data indicated that the parasitoid was not attracted to the M8808 cultivar, a known whitefly‐resistant plant (Rodrigues et al. 2021b), and exhibited largely inactive foraging behavior. Thus, developing and testing cultivars with enhanced resistance to insect herbivory is critical for understanding whether these technologies are compatible with the functional role of natural enemies (Riddick and Simmons 2014).

Since parasitoid behavior can be modulated by cultivar, biological control strategies should consider the impact of the chemical and physical characteristics of host plants on the foraging efficiency and parasitism of *Encarsia* sp. Another relevant approach would be to investigate the use of synthetic volatiles or chemical stimuli derived from *B. tabaci* adults to enhance the attraction of *Encarsia* sp. in the field. Studies indicate that the synergy between host and plant chemical signals may be fundamental for parasitoid attraction (Liu et al. 2019). Therefore, future research should evaluate whether specific compounds emitted by adults can be used to increase the retention and efficiency of *Encarsia* sp. in agricultural systems.

Future research should focus on identifying the specific volatile compounds emitted by soybean cultivars and their influence on the attraction and foraging behavior of *Encarsia* sp. Additional studies could also investigate whether the interaction between plant volatiles and chemical signals released by *B. tabaci* influences the parasitoid's response, potentially modulating its efficiency in biological control. Experiments under field conditions are also essential to assess whether the behavioral patterns observed in the laboratory are maintained in a real agricultural environment, as multiple ecological factors can affect parasitoid decision‐making. It is important to note that key parameters such as parasitism rate, emergence success, and adult longevity are currently being evaluated in complementary studies with specific designs. Although not included here, such parameters are critical for assessing the full biocontrol potential of *Encarsia* sp. and will be addressed in future publications.

Finally, it is important to consider that different species or populations within the genus *Encarsia* may exhibit marked variation in host plant use, sensitivity to plant volatiles, and foraging behavior (Li et al. 2014; Chen et al. 2020; Saranya and Kennedy 2022). This functional diversity can directly influence parasitism efficiency and, consequently, the success of conservation biological control strategies. The recommendation to investigate additional *Encarsia* species is therefore highly relevant, as it may reveal agents with greater responsiveness to specific chemical cues or better adaptation to crop environments.

## 5 Conclusions

The volatiles emitted by the tested soybean cultivars did not significantly influence the attraction of the *Encarsia* sp. parasitoid in the Y‐shaped olfactometer. However, the Bônus cultivar appeared to exert a more attractive effect and promoted a more diversified foraging behavior compared to the whitefly‐resistant M8808 cultivar. These findings highlight that the foraging behavior of *Encarsia* sp. can be modulated by the chemical and physical characteristics of host plants. Such traits represent promising tools for developing innovative biological control strategies aimed at enhancing the efficiency of *Encarsia* sp. in host location and parasitism.

## Author Contributions

**Maria Carolina Farias e Silva:** conceptualization (equal); investigation (equal); writing – original draft (equal). **Raylson Lopes da Silva:** investigation (equal); methodology (equal). **Matheus Monteiro de Santana:** investigation (equal); methodology (equal); software (equal); visualization (equal); writing – original draft (equal). **Daniel Marques Pacheco:** formal analysis (equal); investigation (equal); software (equal); writing – original draft (equal). **Jean Pierre Cordeiro Ramos:** formal analysis (equal); investigation (equal); software (equal); writing – original draft (equal). **Rafael Souza Miranda:** conceptualization (equal); data curation (equal); investigation (equal); software (equal); supervision (equal); writing – original draft (equal). **Jose Bruno Malaquias:** conceptualization (equal); data curation (equal); software (equal); supervision (equal); validation (equal); writing – original draft (equal). **Jenilton Gomes da Cunha:** formal analysis (equal); software (equal); visualization (equal); writing – original draft (equal). **Bruno Ettore Pavan:** conceptualization (equal); formal analysis (equal); software (equal); visualization (equal); writing – review and editing (equal). **Luciana Barboza Silva:** conceptualization (equal); formal analysis (equal); funding acquisition (equal); investigation (equal); project administration (equal); supervision (equal); writing – original draft (equal).

## Conflicts of Interest

The authors declare no conflicts of interest.
